# Cerebral Ischemia Is Exacerbated by Extracellular Nicotinamide Phosphoribosyltransferase via a Non-Enzymatic Mechanism

**DOI:** 10.1371/journal.pone.0085403

**Published:** 2013-12-31

**Authors:** Bing Zhao, Meng Zhang, Xue Han, Xia-Yan Zhang, Qiong Xing, Xu Dong, Qiao-Juan Shi, Peng Huang, Yun-Bi Lu, Er-Qing Wei, Qiang Xia, Wei-Ping Zhang, Chun Tang

**Affiliations:** 1 Department of Pharmacology, Key Laboratory of Medical Neurobiology of Ministry of Health of China, Zhejiang Province Key Laboratory of Neurobiology, Zhejiang University School of Medicine, Hangzhou, Zhejiang, China; 2 State Key Laboratory of Magnetic Resonance and Atomic and Molecular Physics, Wuhan Center for Magnetic, Wuhan Institute of Physics and Mathematics, Chinese Academy of Sciences, Wuhan, China; 3 Department of Physiology, Zhejiang University School of Medicine, Hangzhou, China; 4 Department of Anesthesiology, the First Affiliated Hospital, School of Medicine, Zhejiang University, Hangzhou, China; Albany Medical College, United States of America

## Abstract

Intracellular nicotinamide phosphoribosyltransferase (iNAMPT) in neuron has been known as a protective factor against cerebral ischemia through its enzymatic activity, but the role of central extracellular NAMPT (eNAMPT) is not clear. Here we show that eNAMPT protein level was elevated in the ischemic rat brain after middle-cerebral-artery occlusion (MCAO) and reperfusion, which can be traced to at least in part from blood circulation. Administration of recombinant NAMPT protein exacerbated MCAO-induced neuronal injury in rat brain, while exacerbated oxygen-glucose-deprivation (OGD) induced neuronal injury only in neuron-glial mixed culture, but not in neuron culture. In the mixed culture, NAMPT protein promoted TNF-α release in a time- and concentration-dependent fashion, while TNF-α neutralizing antibody protected OGD-induced, NAMPT-enhanced neuronal injury. Importantly, H247A mutant of NAMPT with essentially no enzymatic activity exerted similar effects on ischemic neuronal injury and TNF-α release as the wild type protein. Thus, eNAMPT is an injurious and inflammatory factor in cerebral ischemia and aggravates ischemic neuronal injury by triggering TNF-α release from glia cells, via a mechanism not related to NAMPT enzymatic activity.

## Introduction

Cerebral ischemia is a neuronal disorder that causes high mortality and disability. Immediately after an ischemic attack, neuronal and non-neuronal cell deaths are induced by events such as energy failure, intracellular calcium overload, excitotoxicity and oxidative stress [1-3]. Subsequently, the release of pro-inflammatory factors triggers inflammation in peripheral and central systems and inflicts additional damage to the neuron [1,4]. As the brain-blood-barrier (BBB) breaks down, the interplay between central and peripheral inflammatory factors further aggravates the damage of an ischemic attack[5].

Nicotinamide phosphoribosyltransferase (NAMPT), also known as pre-B cell enhancing factor (PBEF) [6] or visfatin [7], is located both intracellularly (iNAMPT) and extracellularly (eNAMPT)[8]. NAMPT can be found in various tissues including liver, kidney, skeletal muscle, immune cells and brain [8-10]. The iNAMPT is a key enzyme in the salvaging pathway of nicotinamide adenine dinucleotide (NAD) biosynthesis. NAD plays a vital role in energy metabolism [11,12], and regulates cellular functions by serving as a cofactor for enzymes including sirtuin 1 and poly (ADP-ribose) polymerase-1 (PARP-1) [13,14]. In brain, iNAMPT is primarily expressed in neuron[10,15], and it has been shown that iNAMPT protects the neuron against ischemic injuries through the synthesis of NAD[10,15,16].

The study on eNAMPT has been lagging behind that on iNAMPT. Though lacking a secretion signal, eNAMPT protein has been found secreted by cells including leucocytes [17], hepatocytes [18] and lipocytes [7]. Clinical studies showed elevated level of eNAMPT in serum for patients with diseases including diabetes[19], obesity[20] and cardiovascular diseases[21,22], and it has been reported that higher level of eNAMPT in serum is associated with higher risk of cerebral ischemia[23]. In addition, the NAMPT protein level was elevated in ischemic region [15] and in blood serum [23] after ischemic stroke. Nevertheless, these studies did not differentiate the respective change of eNAMPT versus iNAMPT in ischemic brain, and thus the exact role of eNAMPT in the related brain diseases has not been established.

In this study, we showed that eNAMPT level became elevated in ischemic brain region after MCAO and reperfusion in rats, and the protein may have come from blood circulation through a damaged BBB. Furthermore, we found that eNAMPT exacerbated ischemic neuronal injury upon triggering the release of TNF-α from glia cells, via a mechanism not related to the enzymatic activity of NAMPT. 

## Materials and Methods

### Ethics statement

Purchased from Experimental Animal Center of Zhejiang Academy of Medicine Sciences, 112 male Sprague–Dawley rats (250–300 g, 3 month’s old) and around 100 newborn rats were incorporated. All procedures were carried out in accordance with the recommendations in the Guide for the Care and Use of Laboratory Animals of the National Institutes of Health. The experimental protocols were approved by the Ethics Committee of Laboratory Animal Care and Welfare, School of Medicine, Zhejiang University. All animals were anesthetized under 10% chloral hydrate before sacrifice, and efforts were made to minimize suffering.

### Preparation of recombinant human NAMPT protein

With a hexa-histidine tag appended to the C-terminus, recombinant human NAMPT and H247A mutant were expressed in E. coli BL21 (DE3) cells, and were purified with Hi-Trap nickel affinity and S-200 size-exclusion columns on ÄKTA Purifier system (GE healthcare). Protein samples were spin concentrated and exchanged to PBS buffer in Amicon Ultra (Millipore). Purified proteins were confirmed by electrospray mass spectrometry (Bruker Daltonics). The active-site mutant of NAMPT protein, H247A, has essentially no enzymatic activity as assessed by NMR spectroscopy [24].

To exclude the contaminations from protein expression and purification from bacterial that may induce *in vivo* and *in vitro* responses, we expressed and purified a recombinant signaling protein named enzyme I (short as EI, Uniprot accession code P08839), inform bacterial phosphotransferase system (PTS), as an additional control. EI has the similar molecular weight with NAMPT (56003.6 Da for His-tagged NAMPT and 63562 Da for EI, and both proteins are dimeric).

### Middle-cerebral-artery occlusion (MCAO) and intracerebroventricular administration of NAMPT protein

Rats were anesthetized with an i.p. injection of chloral hydrate (400 mg/kg). MCAO was performed as described [25]. A polyethylene tube was inserted into the right femoral artery to measure arterial partial pressure of oxygen (pO_2_), arterial partial pressure of carbon dioxide (pCO_2_) and arterial blood pH (Blood Gas Analyzer ABL 700, Radiometer, Copenhagen, Denmark). Blood pressure was measured by a noninvasive tail cuff connected to a PowerLab system (AD Instruments Pty Ltd., Castle Hill, NSW, Australia). Blood glucose was monitored by one touch basic blood glucose monitoring system (Lifescan Inc., USA).

Regional cerebral blood flow (rCBF) was recorded using a laser Doppler flowmeter (ML191, AD Instruments Pty. Ltd.). A 2.0 mm diameter burr hole was carefully drilled in the skull at 2.0 mm posterior to bregma and 6.0 mm lateral from the mid-line on the ipisilateral to the MCAO. The hole was not penetrated through the skull, so that very thin skull remained intact. An acrylic guide cannula (OD, 2 mm; ID, 1 mm) was placed perpendicularly on the surface of the intact skull and fixed by using Loctite 411 instant adhesive and Insta-set accelerator. One end of the guide cannula has a transparent septum, which directly comes in contact with the skull. The guide cannula was filled with distilled water. A laser Doppler flowmetry probe (1 mm diameter) connected to a laser Doppler flowmeter was inserted into the guide cannula until the tip reached the septum of the cannula to allow continuous measurement of the regional cerebral blood flow (rCBF). The steady-state baseline of rCBF value before ischemia was defined as 100%. Only rats with rCBF reduced by more than 70% during occlusion and recovered by more than 80% during reperfusion were included. Rectal (core) temperature was recorded and maintained at 36.5-37.5 °C with a heating pad (FHC, Bowdoinham, ME, USA) during the surgery.

For MCAO, a midline incision was made in the neck, the right external carotid artery (ECA) and internal carotid artery (ICA) were carefully exposed, and a 3-0 nylon suture was inserted into ICA from ECA to block the origin of the middle cerebral artery. Sham-operated rats underwent the same surgery except for inserting the intraluminal suture. 30 minutes after occlusion, the suture was withdrawn to allow reperfusion, which was ascertained by restoration of rCBF. Then the incision was closed, and the laser Doppler flowmetry probe and polyethylene tube in femoral artery were removed. Rats were kept in a warm box for about 2-3 h to maintain body temperature until they woke up from anesthetization.

For intracerebroventricular administration, a metallic cannula was implanted into the lateral ventricle (0.9 mm posterior to bregma, 1.4 mm from the mid-line, 3.4 mm below brain surface). After one week of recovery, either 4 μl saline, or 4 μg albumin, or recombinant human NAMPT protein (either wild type or H247A mutant) solution, or EI protein solution prepared in 4 μl saline was administrated through the cannula at three time points: 12 h before MCAO, immediately after MCAO, and 12 h after MCAO. After various reperfusion times, neurological deficit score and infarct volume were evaluated as described [25].

### Behavioral assessment and infarct volume analysis

Neurological deficit scores were evaluated by a researcher who is blind to the groups by following the reported protocol [26]: 0, no deficit; 1, flexion of the contralateral forelimb upon lifting of the whole animal by the tail; 2, decrease of thrust toward the contralateral plane; 3, circling to the contralateral side.

For the measurement of infarct volume, brains were quickly removed and sliced into 2 mm thick-slices in brain matrix. The brain sections were incubated in 0.05% 2,3,5-triphenyltetrazolium chloride (TTC) in phosphate-buffered saline for 30 min at 37 °C, and then transferred into 4% formaldehyde solution for fixation. The unstained area was identified as infarct area. The infarction area and the area of ipsilateral hemisphere in each brain sections were measured by ImageTool 2.0 software (University of Texas Health Science Center, San Antonio, TX, USA). Infarct volume was calculated by summing up the volumes in the six slices, and presented as the percentage of infarction in the summed area of hemisphere in the respective figures. 

### Determination of the NAMPT protein level in cerebral-spinal fluid (CSF)

After MCAO and reperfusion, rats were anesthetized by i.p. injection of chloral hydrate (500 mg/kg). The fur over the caudal head was removed. The rat brain was fixed on stereotaxic instrument, and the height of brain was adjusted until the occipital bone was almost horizontal to the table and the body was lying at a 45° angle to the head. A soft area in the midsagittal plane approximately 12 mm caudal to the base of ears allowed for the collection of CSF. A needle (22 gauge) was carefully inserted into this soft surface until disrupt through the dura into the cisterna magna. Subsequently, about 100 μl CSF was carefully collected and samples with visible evidence of blood were excluded from further analysis. NAMPT protein was determined by using a NAMPT (Visfatin/PBEF) ELISA Kit (Adipogen).

### Immunoblotting

Total protein from brain tissues was extracted by following the manufacturer’s instruction of Cell and Tissue Protein Extraction Kit (Kangchen Biotechnology). Briefly, the tissue was homogenized in radio immunoprecipitation assay buffer. The homogenate was centrifuged at 12,000 g for 30 min at 4°C, and then quantified by using Coomassie brilliant blue method. Protein samples (60 μg) were separated by 10% SDS page gel and blotted onto nitrocellulose membranes (Invitrogen). Blots were incubated overnight at 4 °C either with rabbit polyclonal antibody against NAMPT (1:1000; Bethyl) and mouse monoclonal antibody against glyceraldehydes-3-phosphate dehydrogenase (GAPDH, 1:5000; Kangchen Biotechnology), or with mouse monoclonal antibody against His-tag (1:2000; Abmart) and rabbit monoclonal antibody against GAPDH (1:5000, Kangchen Biotechnology). Subsequently, the blots were reacted with IRDye-700CW goat anti-rabbit IgG (1:5000; Rockland), and IRDye-800CW anti-mouse IgG (1:5000; Rockland). The image was captured by Odyssey infrared imaging system (Li-Cor Bioscience). 

### Reverse transcription-polymerase chain reaction (RT-PCR)

Total RNA from brain tissues was extracted using Trizol reagents (Life Technologies) according to the manufacturer’s instruction, and quantified by using spectrophotometry (Smart+Spec^TM^ 3000, BIO-RAD). The specificity of the oligonucleotide primers was verified using the program BLASTN. For cDNA synthesis, 2 μg total RNA was mixed with 1 mM dNTP, 0.2 μg random primer, 20 U RNasin, 200 U M-MuLV reverse transcriptase in 20 μl total volume. The mixture was incubated at 42°C for 60 min, and then heated to 72 °C for 10 min to inactivate the reverse transcriptase. As negative control, the reaction was performed at the absence of reverse transcriptase. PCR was performed on an Eppendorf Master Cycler (Eppendorf, Hamburg, Germany) as following: 1 μl cDNA mixture was reacted in 20 μl of the reaction buffer containing 1.5 mM MgCl_2_, 0.2 mM dNTPs, 20 pM primer, 1 U Taq DNA polymerase. The reactions were initially heated at 94°C for 4 min; then at 94°C for 30 s, 52°C for 30 s and 72°C for 45 s, totally 30 cycles; finally stopped at 72°C for 7 min. 10 μl PCR products were separated by 2% agarose gel electrophoresis and analyzed by UVP gel analysis system (Bio-Rad, Richmond, CA, USA). The expression of NAMPT mRNA was expressed as ratios compared with β-actin. The primers for PCR comprise NAMPT forward (5'-TTGCTGCCACCTTACCTTAG-3'), NAMPT reverse (5'-GAGCTTTTTGTCACCTTCCC-3'), β-actin forward (5’-TGACGTTGAC ATCCGTAAAG-3’) and β-actin reverse (5’- ACAGTGAGGCCAGGATAGAG-3’). The PCR products are 386 and 190 bp for NAMPT and β-actin, respectively.

### Immunochemistry and immunofluorescence

For immunochemistry, 10 μm-thick cryo-brain sections were incubated with 3% hydrogen peroxide and blocked with 5% goat serum. The brain sections were incubated at 4 °C overnight with mouse monoclonal antibody against His-tag (1:1000; Abmart). After washing, the brain sections were incubated with biotinylated goat anti-mouse IgG (1:200; Vector Labs) and streptavidin-horseradish peroxidase conjugate (1:200; Vector Labs). The sections were exposed for 2 min to 0.05% 3, 3-diaminobenzidine and 0.03% H_2_O_2_. A light microscope (Olympus BX51, Japan) was used for image capture.

For immunofluorescence, brain sections or cells were blocked with 5% donkey serum, and incubated overnight at 4°C with one or two antibodies: rabbit polyclonal antibody against NAMPT, mouse monoclonal antibodies against NeuN (1:200; Chemicon), mouse monoclonal antibodies against GFAP (1:800; Chemicon), or goat polyclonal antibody against Iba-1 (1:500; Abcam). The sections were incubated with the mixture of Cy3 and FITC conjugated anti-rabbit and anti-mouse/goat IgG antibody (1:200; Chemicon), and were mounted with an anti-fade medium containing DAPI (Invitrogen). The cortex of layer III-IV in the boundary zone and ischemic core, as well as the relative area in the sham mouse brain was observed under a fluorescent microcopy (Olympus BX51).

### Cell culture and oxygen-glucose-deprivation (OGD)

Neuron-glia mixed culture was prepared from the cerebral cortex of newborn rats, and was cultured in plating medium containing 80% high glucose DMEM, 10% fetal bovine serum, 10% horse serum, 2 mM glutamine, 100 units/ml penicillin, 100 μg/ml streptomycin. 24 h after isolation, the medium was changed to feeding medium containing 95% high glucose DMEM with 5% horse serum, 0.01% N2 and 0.04% B27. The medium was changed every 3 days. Around 28% of the cultured cells were neurons, 7% of the cultures were microglia cells and the majority of the remaining cells are astrocytes.

To inhibit the growth of glial cells in cultured neuron cells, 10 µM cytosine arabinoside was applied on the third day of culture. More than 95% of the cultured cells were neurons, as assessed by immunofluorescent staining for MAP2 (1:500; Millipore).

For OGD, cells were washed 3 times and incubated with glucose-free Earle’s solution. The cells were incubated under 95% N_2_ and 5% CO_2_ at 37°C for 1 h. Control cells were placed in Earle’s solution with 25mM glucose and were incubated under normal culture conditions for 1 h. After OGD, the cells were incubated in regular culture medium under 5% CO_2_ and 95% air to allow recovery. 

### Analysis of the Percentage of Necrotic Cells and Necrotic Neuron Cells

To assess cell death, 24 h after OGD and recovery, cells grown on coverslips were stained with 10 μg/ml of propidium iodide. Cells were then washed and fixed with 4% paraformaldehyde, and immunofluorescence stained with NeuN (to stain neurons). After mounted with an anti-fade medium containing DAPI, cells were imaged under a fluorescent microcopy (Olympus BX51). The percentage of necrotic cells was determined as the number of PI-positive cells (stained red) over the total number of cells; the percentage of necrotic neuron cells was determined as the number of PI and NeuN double positive cells over the number of NeuN-positive cells. For each sample analysis, a total of ~5000 cells were counted by a researcher who is blind to the different groups.

### Assessment of cell viability and lactate dehydrogenase (LDH) release

24 h after OGD and recovery, cell viability was evaluated with MTT assay. Briefly, cells were incubated in 0.5 mg/ml MTT for 4 h at 37°C. After washing, 100 µL DMSO was added and incubated for 10 min. The 96-well plate was read at 490 nm using a microplate reader (Elx800, Bio-Tek Instrument). Results were reported as the percentages of control. LDH released into medium was measured using LDH detection kit (Jianchen) according to the manufacturer’s instruction. 

### Total NAD (tNAD) measurement

Total NAD level in brain was determined by using a NAD^+^/NADH quantification kit (BioVision). Briefly, the cortex in the contralateral, boundary zone and ischemic core was homogenized in NADH/NAD extraction solution, and total NAD level was measured in the presence of NAD cycling mix and NADH developer. Optical density was read at 450 nm every 30 minutes for 4 hours. The total NAD level was expressed as pmol/mg tissue.

### TNF-α measurement

TNF-α level was determined using rat TNF-α Quantikine ELISA kit (R&D Systems). Briefly, the supernatant of cell culture was collected 24 h after OGD and recovery and particles were removed by centrifugation (10000 g for 10 min). 50 μl sample together with 50 μl assay diluent RD1-41 was added to the well and incubated for 2 h. After wash, 100 μl rat TNF-α conjugate, 100 μl substrate solution and 100 μl stop solution was added to each well. The resulting optical density was read at 570 nm within 30 min. 

### Statistical analysis

All data are expressed as mean ± SD. Values from different groups were compared using one-way ANOVA. Statistical analysis was done in Prism 4.0 (GraphPad Software). *P*<0.05 was considered statistically significant.

## Results

### eNAMPT protein level increased in brain upon cerebral ischemia

**Figure 1:**
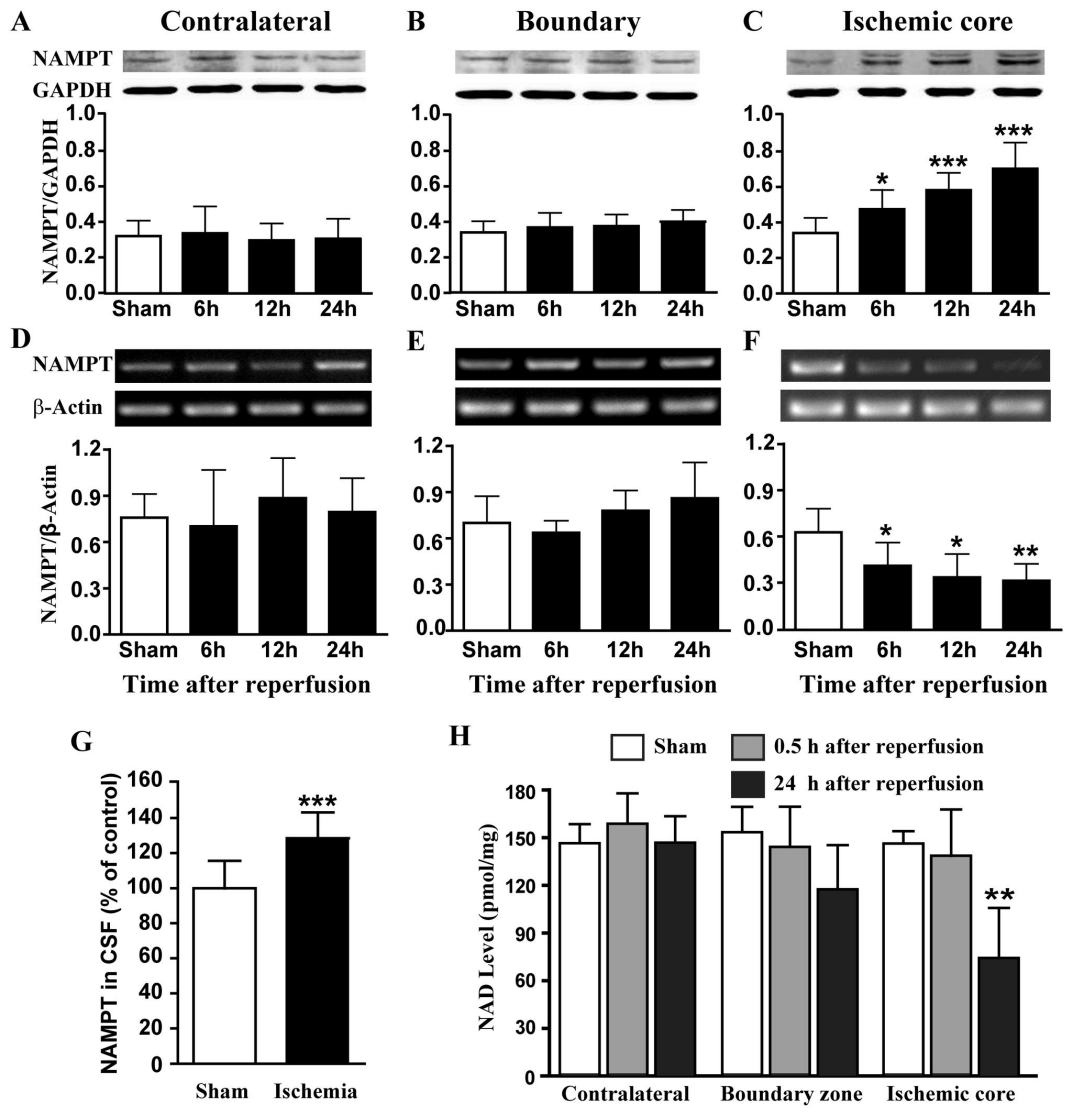
NAMPT and NAD levels in rat brain after ischemia. (**A**-**C**) NAMPT protein expression level was measured by using Western blot in the contralateral (A), boundary zone (B) and ischemic core (C). N=8. (**D**-**F**) NAMPT mRNA expression level was measured by using RT-PCR in the cortex of ischemic core. N=5. (**G**)NAMPT protein level in cerebral-spinal fluid was measured after 30 min MCAO and 24 h reperfusion. N=10. (**H**) NAD level was measured in rat brain after cerebral ischemia. N=4. *P<0.05, **P<0.01, ***P<0.001, compared with sham, One-way ANOVA.

After cerebral ischemia, total NAMPT protein level in the ischemic core increased by 39%, 70% and 106% (Figure 1C), whereas NAMPT mRNA level in the ischemic core decreased by 35%, 47% and 50% at 6, 12 and 24 h after reperfusion, respectively (Figure 1F). Both NAMPT protein and mRNA expression levels remained constant in the contralateral cortex and boundary zone (Figure 1A, 1B, 1D and 1E). The NAMPT protein level in CSF increased by 28.5 % at 24 h after reperfusion (Figure 1G). NAD, the product of NAMPT, remained unchanged at 0.5 h after reperfusion and decreased by 49% at 24 h after reperfusion in the ischemic core, while remained constant in the boundary zone and contralateral cortex regions at 0.5 h and 24 h after reperfusion (Figure 1H). 

**Figure 2:**
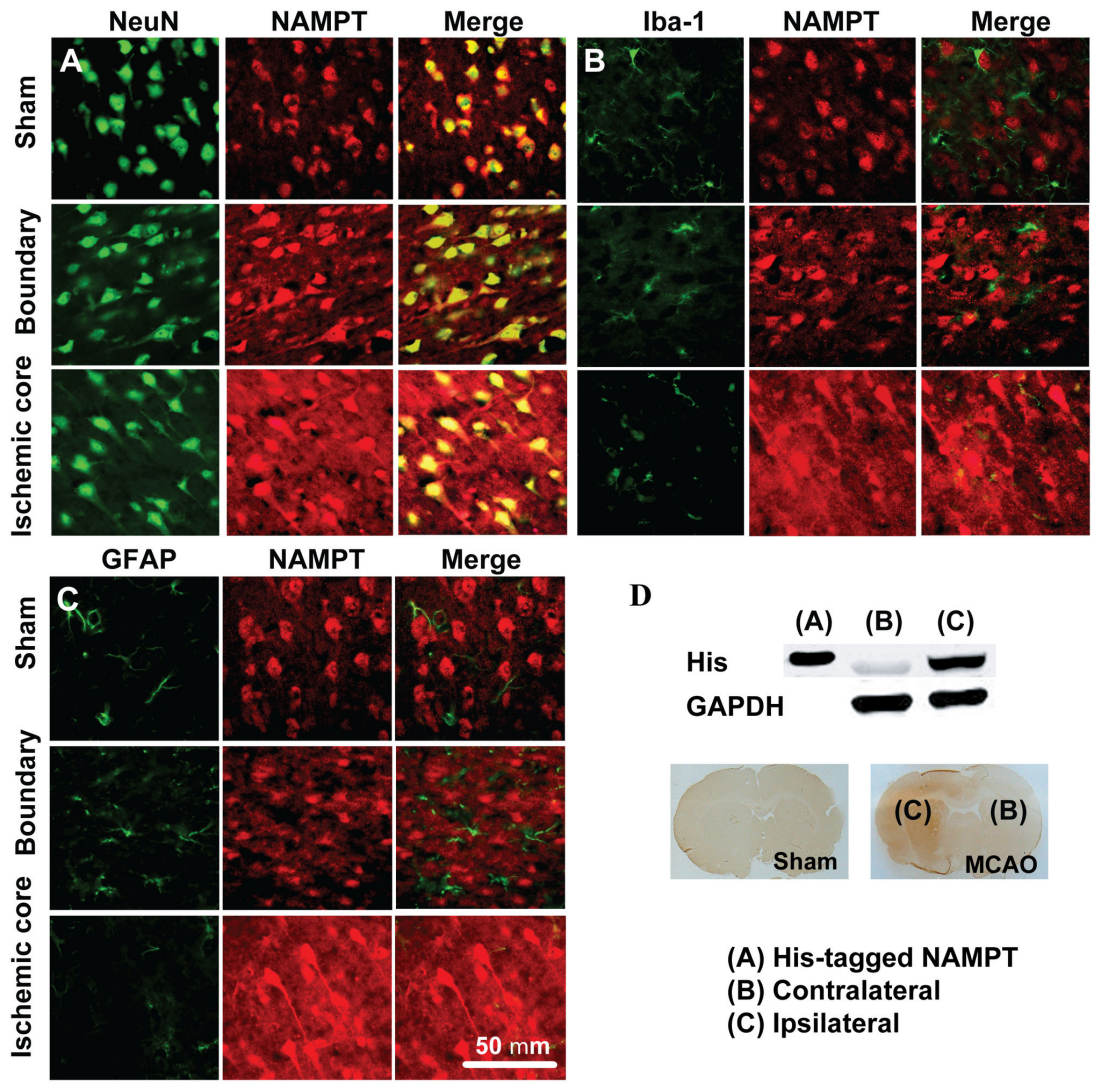
Distribution of NAMPT protein in rat brain after MCAO. (**A**-**C**) Representative images of double immunofluorescent staining of NeuN (for neuron) and NAMPT (A), Iba-1 (for microglia) and NAMPT (B), GFAP (for astrocyte) and NAMPT (C) in rat brain after 30 min MCAO and 24 h reperfusion. Immunofluorescent staining was repeated on three rats. (**D**) Using Western blot (upper panel) and immunochemistry staining (lower panel), the i.v. injected His-tagged NAMPT was detected in the ischemic brain area after 30 min MCAO and 24 h reperfusion.

NAMPT protein was found mainly expressed in neuron (Figure 2A), and expressed in certain Iba1-positive microglia cells after MCAO and reperfusion (Figure 2B), but not in astrocyte (Figure 2C). In addition, the fluorescence signal evenly increased in the areas in addition to the NAMPT-positive cell bodies, which can be attributed to an elevated level of eNAMPT (Figure 2A-2C). To investigate the source of the increased eNAMPT in ischemic region, rats were intravenously injected with 10 μg His-tagged NAMPT immediately after cerebral ischemia. The recombinant protein was detected in the ischemic core but not in the contralateral cortex 12 h after reperfusion (Figure 2D). 

### Administration of recombinant NAMPT exacerbated ischemic brain injury

**Figure 3:**
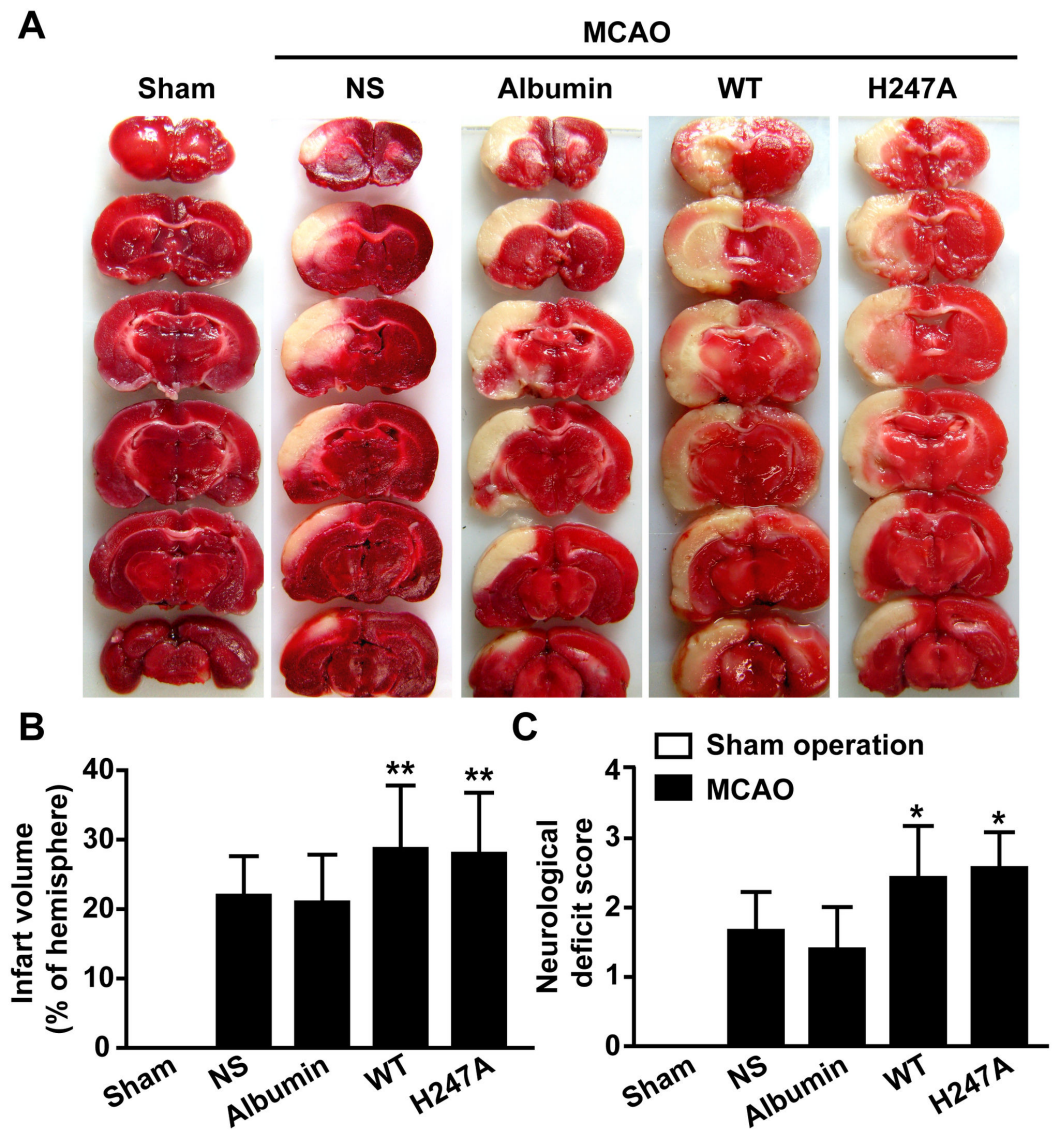
Recombinant NAMPT protein aggravated MCAO-induced brain injury. The rats were i.c.v. injected with 4 μg albumin, wild type NAMPT (WT) or H247A NAMPT (H247A) at 12 h interval for three times with the first injection 12 h before MCAO. (**A**) Representative brain sections stained with TTC. (**B**) Statistical analysis of the infarct volume. (**C**) Neurological deficit score evaluated before sacrifice. N=6, *P<0.05, **P<0.01, compared with NS, One-way ANOVA. NS represents normal saline.

Intracerebroventricular administration of NAMPT protein (either wild type protein or H247A mutant) did not affect the physiological parameters of the rats monitored (Table S1). However, administration of either wild type protein or H247A mutant proteins resulted in a significant increase in the infarctvolume 24 h after reperfusion (from 22.0 ± 2.0 % to 28.7 ± 3.6% and 28.0 ± 3.5 %, for wild type or H247A mutant respectively) (Figure 3A and 3B). Moreover, the NAMPT proteins caused a significant increase of neurological deficit score (Figure 3C). As a control, administration of human albumin had no effect on infarct volume or neurological deficit score after MCAO (Figure 3A-3C). As a second control by administrating, recombinant protein prepared from the same bacteria host cells as NAMPT, EI protein exerted no effect on infarct volume or neurological score after MCAO (Figure S1 and Table S2). 

### NAMPT aggravated OGD-induced neuronal injury in neuron-glial mixed but not in cultured neuron

**Figure 4:**
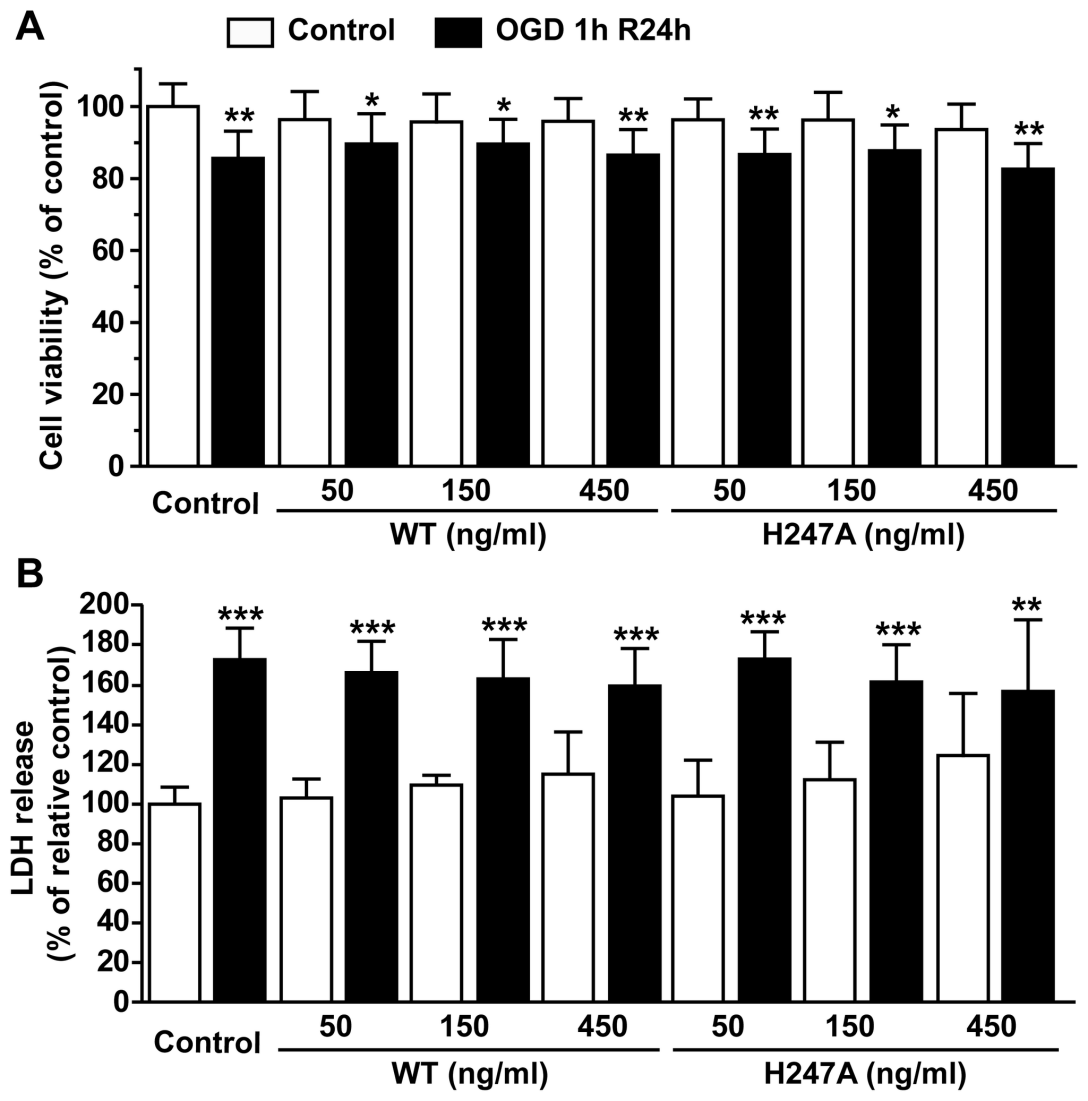
The effect of recombinant NAMPT protein on primary cultured neuron cell injury induced by OGD and reperfusion. Wild type NAMPT (WT) or H247A NAMPT (H247A) was applied 30 min before OGD. (**A**) Using MTT assay, cell viability was determined after the treatment of NAMPT or combined treatment with NAMPT and OGD. (**B**) LDH in medium was determined after the treatment of NAMPT or after combined treatment with NAMPT and OGD. N=12. *P<0.05, **P<0.01, ***P<0.001, compared with relative control, One-way ANOVA.

Using MTT assay, we found that application of wild type or H247A NAMPT protein (50, 150 and 450 ng/ml) did not affect cell viability (Figure 4A) and LDH release (Figure 4B) of cultured neuron cells under normal or OGD conditions. 

**Figure 5:**
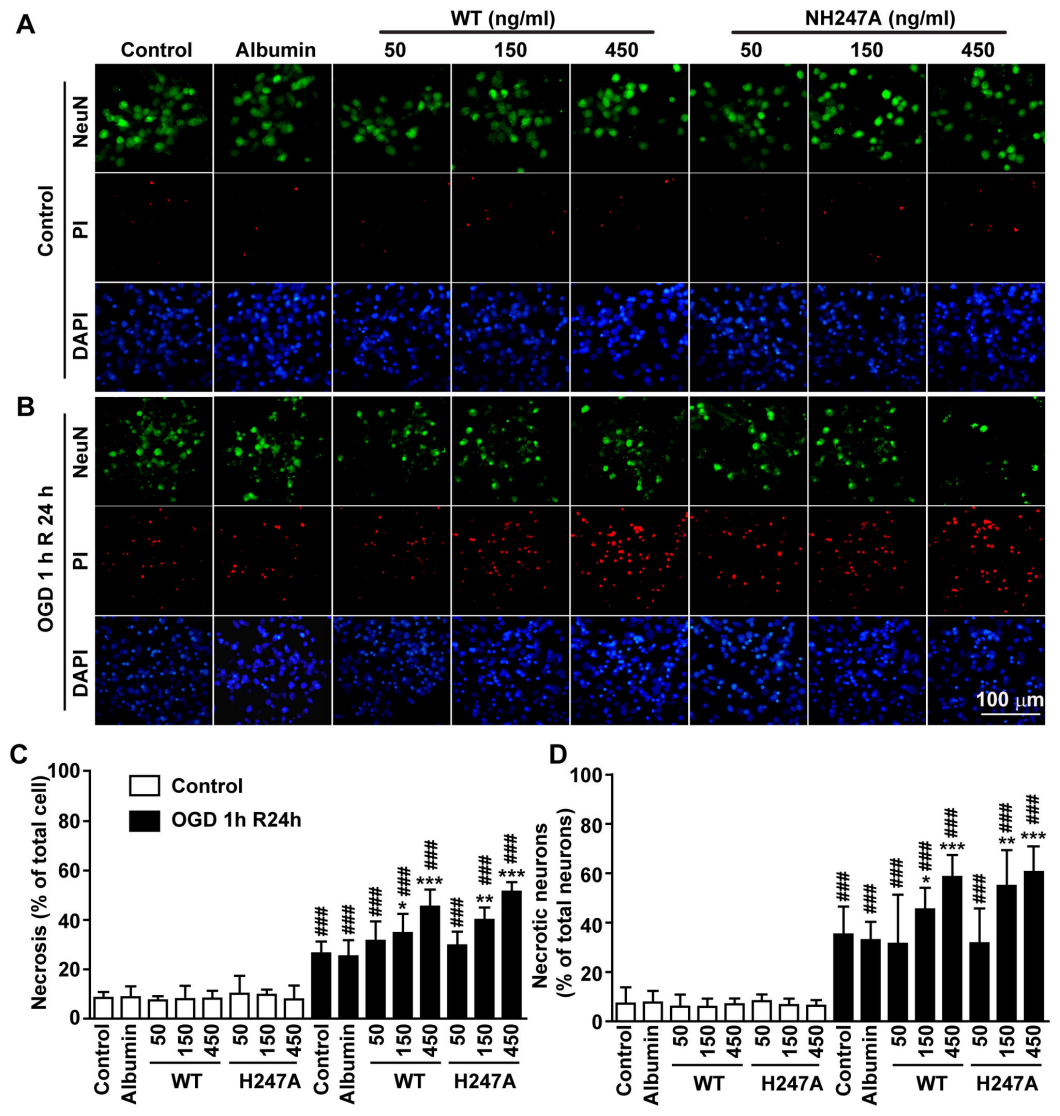
The effect of recombinant NAMPT protein on OGD-induced cell necrosis in neuron-glial mixed culture. Wild type NAMPT (WT) or H247A NAMPT (H247A) protein was applied 30 min before OGD. (**A** and **B**) Representative images of triple immunofluorescence staining of NeuN (green, neuron specific marker), PI (red, for necrotic cells) and DAPI (blue, for all cells) under normal condition (A) or after OGD and recovery (B). (**C**) Percentage of necrotic cells in total cells (PI-positive cell number over DAPI-positive number). N=6. (**D**) Percentage of necrotic neurons in total neuron cells (PI/NeuN double positive cell number over NeuN-positive cell number). N=6. *P<0.05, **P<0.01, ***P<0.001, compared with the control of OGD, One-way ANOVA. ^###^ *P*<0.001, compared with the control of control, One-way ANOVA.

In neuron-glial mixed culture, when without OGD, the percentage of necrotic cell and of necrotic neuron cells was 6.9±2.3% and 7.9± 2.4%, respectively (Figure 5A, 5C and 5D). Application of wild type NAMPT or H247A mutant (50, 150 and 450 ng/ml) did not affect the percentage of necrotic cells or necrotic neuron cells (Figure 5A, 5C and 5D). After OGD for 1 h and recovery for 24 h, the percentage of necrotic cells and of necrotic neuron cells increased to 24.0±3.4% and 36.3±5.4%, respectively (Figure 5B-5D). Application of either wild type NAMPT or H247A mutant (50, 150 and 450 ng/ml) further increased the percentage of necrotic cells and of necrotic neurons, in a concentration-dependent manner (Figure 5B-5D). In comparison, application of 450 ng/ml human albumin (Figure 5A-5D) and recombinant EI protein (450 ng/ml) (Figure S2) did not further increase cell necrosis. 

### TNF-α is involved in NAMPT-induced neuronal injury in neuron-glia mixed culture

**Figure 6:**
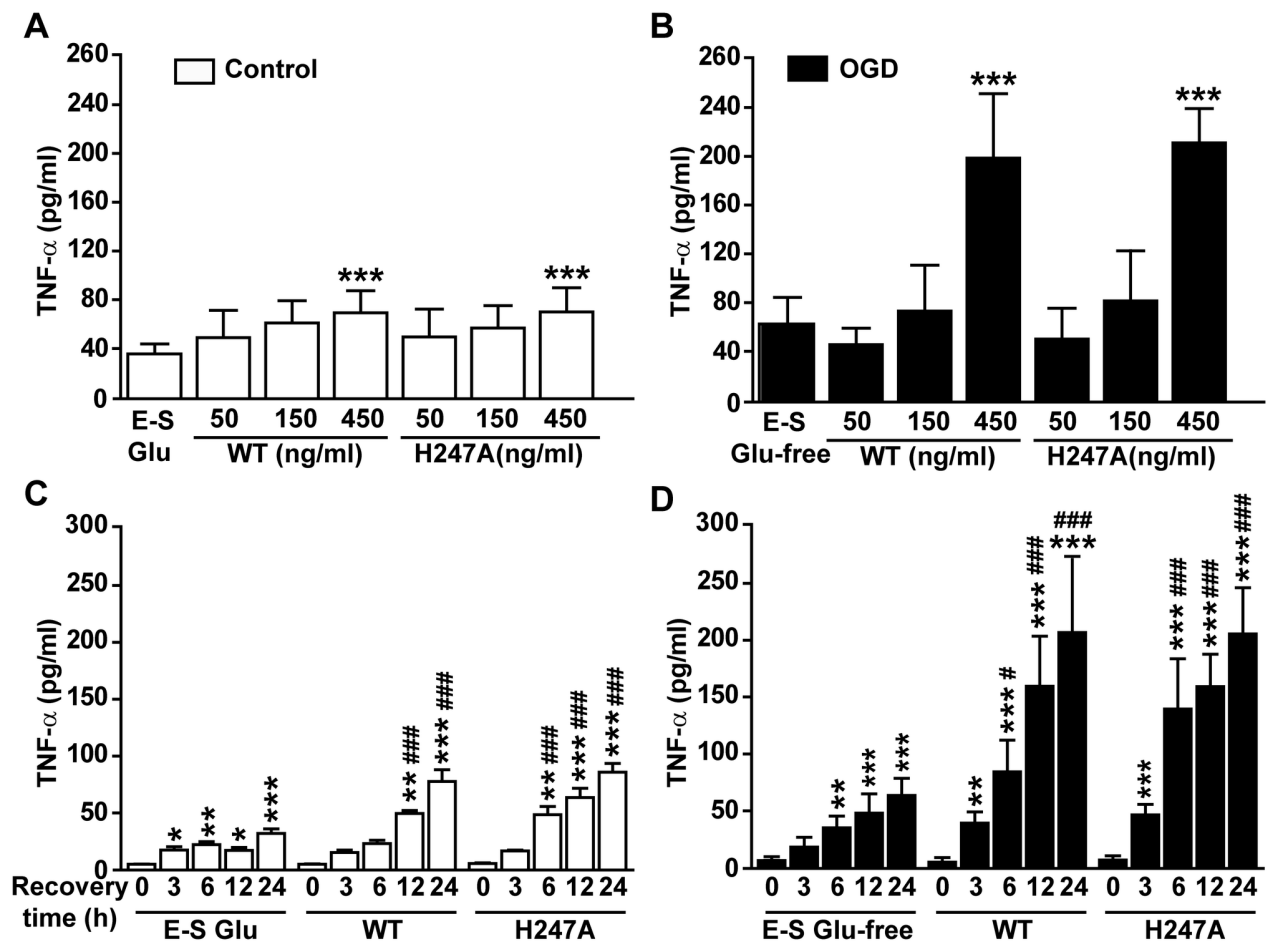
The effect of recombinant NAMPT on ischemic-induced TNF-α release in primary neuron-glial mixed culture. Wild type NAMPT (WT) or H247A NAMPT (H247A) was applied 30 min before OGD. (**A** and **B**) TNF-α in the medium was determined at 24 h after NAMPT treatment (A) or after combined treatment with OGD (B). N=6. ***P<0.001, compared with E-S Glu (A) or E-S Glu-free (B), One-way ANOVA. (**C** and **D**) TNF-α in the medium was determined at 3, 6, 12 or 24 h after the treatment of NAMPT (C) or after combined treatment with NAMPT and OGD (D). N=5. *P<0.05, **P<0.01, ***P<0.001, compared with “0” of E-S Glu (C) or E-S Glu-free (D), One-way ANOVA. ^***#***^ *P*<0.05, ^##^ *P*<0.01, ^###^ *P*<0.001, compared with 0 h recovery in each group, One-way ANOVA. E-S is Earle’s solution; Glu is glucose.

Without OGD, NAMPT induced release of a small amount of TNF-α in neuron-glial mixed culture. 24 h after the application of 450 ng/ml wild type NAMPT or H247A mutant, TNF-α level in medium was elevated from 36.4± 9.4 pg/ml to 70.1±15.5 pg/ml and 70.8±17.3 pg/ml, respectively (Figure 6A). After OGD 1 h and recovery for 24 h, TNF-α level was increased to 63.3±21.9 pg/ml. Under such ischemic condition, application of 450 ng/ml NAMPT protein propelled TNF-α level to a much higher level, 199±68 pg/ml and 212±32 pg/ml for wild type and H247 mutant respectively (Figure 6B). The effect of NAMPT on TNF-α release was accumulative within 3-24 h window we observed (Figure 6C and 6D). 

**Figure 7:**
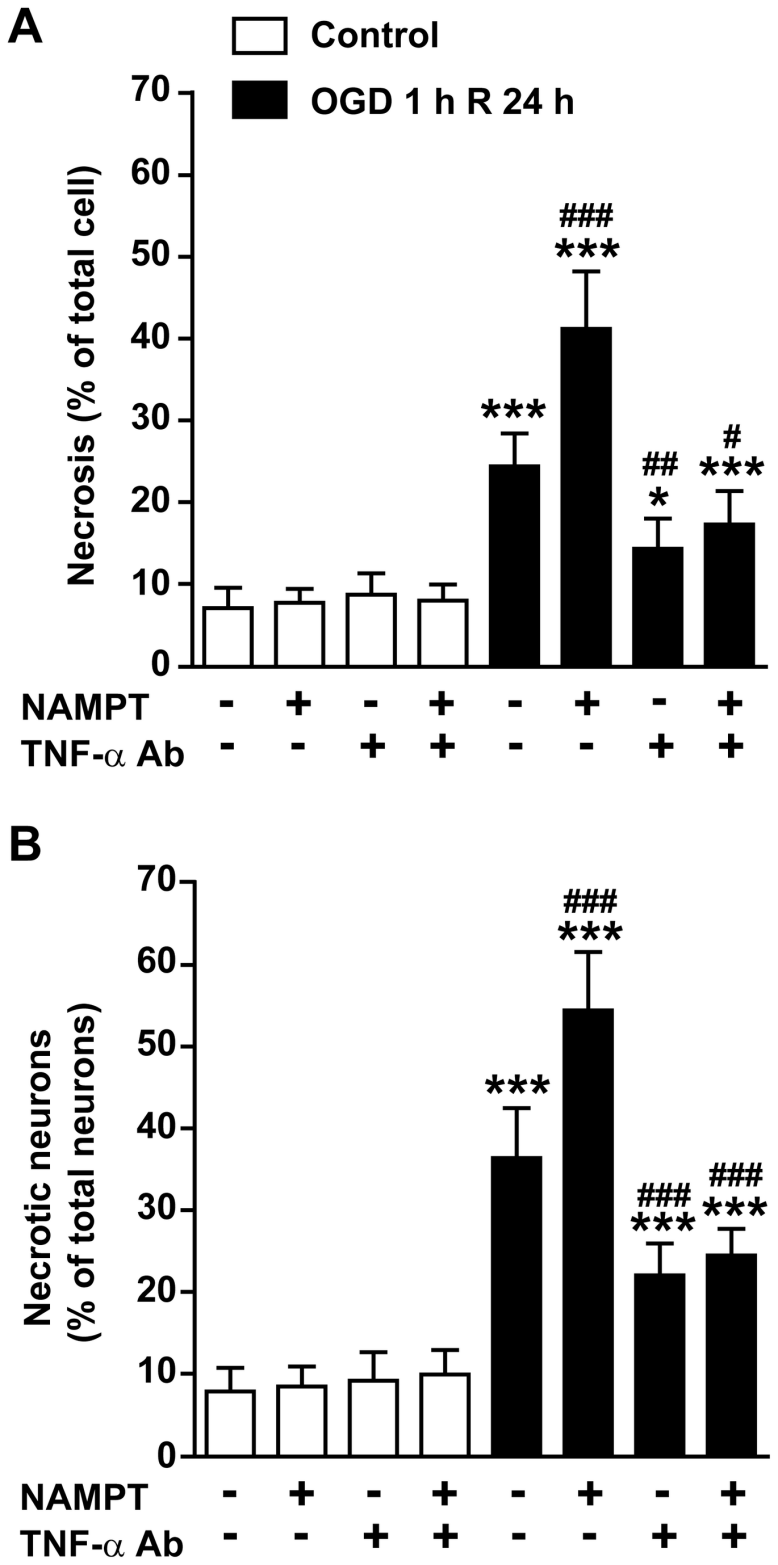
TNF-α neutralizing antibody protected neuronal injury induced by OGD and NAMPT in primary neuron-glial mixed culture. NAMPT and TNF-α neutralizing antibody (5μg/ml, TNF-α Ab) was applied 30 min before OGD. (**A**) The percentage of necrotic cells in total cells determined by using PI and DAPI staining. (**B**) The percentage of necrotic neuron cells determined by using NeuN, PI and DAPI staining. N=5-6. *P<0.05, ***P<0.001, compared with no NAMPT no TNF-α Ab in control, One-way ANOVA. ^***#***^ *P*<0.05, ^##^ *P*<0.01, ^###^ *P*<0.001, compared with no NAMPT no TNF-α Ab in OGD, One-way ANOVA.

To further determine whether TNF-α is involved in the process of NAMPT-aggravated OGD-induced neuronal injury, TNF-α neutralizing antibody (R&D System, Minneapolis, MN, Goat anti-rat polyclonal antibody) was utilized. TNF-α neutralizing antibody (5 μg/ml) had no effect on cell necrosis under normal conditions (Figure 7A and 7B). However, the antibody significantly decreased OGD-induced cell necrosis and neuronal necrosis (Figure 7A and 7B). Importantly, TNF-α neutralizing antibody reverted the effect of recombinant NAMPT protein on cell necrosis and neuronal necrosis after OGD and reperfusion (Figure 7A and 7B).

## Discussion

We have shown that eNAMPT level became significantly elevated in ischemic brain region, which can be traced at least in part to peripheral blood circulation across a damaged BBB. Previous reports showed that overall NAMPT protein level increased in ischemic core region [15] and in blood serum [23] after ischemic stroke. However, these studies did not dissect the respective change of eNAMPT level. Our evidences for an elevated eNAMPT level in brain mainly include the increase of NAMPT level in the CSF and the appearance of recombinant NAMPT in ischemic brain after MCAO. The appearance of NAMPT protein in the areas in addition to cell bodies may also imply an elevation of eNAMPT. On the other hand, the decrease of NAMPT mRNA level may cause a decrease of NAMPT protein expression level and accordingly the iNAMPT protein level. Thus, the decrease of NAD levels can be resulted from a combination of factors, including the decrease of iNAMPT, the reduction of enzymatic activity of iNAMPT, and an increase of NAD consumption by NAD-dependent enzymes such as PARP-1 [27] after cerebral ischemia. 

The increase of eNAMPT in ischemic brain may partly originate from blood circulation, as a large amount of recombinant i.v. injected His-tagged NAMPT could be detected in the ischemic brain region using anti-His antibody. It is known that serum eNAMPT can be secreted from many types of cells including adipose cells[7], hepatic cells[18] and leucocytes[17], and its concentration can reach hundreds of ng/ml[28]. Clinical studies have showed that eNAMPT in serum increased significantly for patients with diseases such as diabetes[19], obesity[29] and atherosclerosis[30,31], who have a higher risk of ischemic stroke. As such, eNAMPT could be one of the factors that responsible for the cross-talking between peripheral and central system after cerebral ischemia. Granted, other sources may also contribute to the increasing eNAMPT level in ischemic brain, for example, from infiltrated neutrophils [17] entering the brain after cerebral ischemia[5], or from dead neurons upon cell lysis[9].

We further showed that eNAMPT exacerbated neuronal ischemic injury, both *in vivo* and *in vitro*. Therefore, the role of eNAMPT is exactly opposite to that of iNAMPT in neuron. It has been reported that iNAMPT protects neurons against ischemic brain injury[10], via several mechanisms, including the synthesis of NAD, activation of SIRT1-MAPK signal pathway[15], induction of autophagy [32], and maintenance of the function of mitochondria[16]. Administration of NAD also protects the neuron against ischemic injury and excitotoxicity [33,34]. For eNAMPT, even though there have been studies indicating that eNAMPT mediates inflammatory responses in peripheral system[35,36], our study provides the first direct evidence that eNAMPT has an injurious effect on cerebral ischemia. 

We have further shown that the injurious effect of eNAMPT on cerebral ischemia was mediated by TNF-α released from glial cells. The reported protective function of iNAMPT is performed within neuron cells [15,16,32]. In comparison, eNAMPT exacerbated ischemic neuronal injury only in glia-neuron mixed culture, but not in pure neuron culture, indicating the participation of glia cells in the process. Furthermore, we showed that eNAMPT triggered the release of TNF-α, whereas TNF-α neutralizing antibody attenuated NAMPT-enhanced neuronal injury after OGD and reperfusion, thus implicating TNF-α in the injurious process of eNAMPT on cerebral ischemia. TNF-α is an inflammatory factor that can be released by glial cells upon cerebral ischemia, and can induce ischemic injury[37,38]. NAMPT-triggered cytokine release including TNF-α has been reported in peripheral diseases [24,35,39]. Interestingly, NAMPT induced a small amount of TNF-α release and did not induced neuronal death when there is no OGD, but induced a large amount of TNF-α release and enhanced neuronal death upon OGD. These findings suggest that some other factors are released from glia cells after OGD amplifying the effect of NAMPT.

We have also demonstrated that the injurious effect of NAMPT was exerted via a mechanism not related to its enzymatic activity. NAMPT, as the name indicates, is an enzyme that catalyzes the synthesis of NAD. It has been previously shown by Li et al that NAMPT promoted macrophage survival via a mechanism not related to NAD synthesis[40]. Using an H247A mutant of NAMPT that possesses essentially no enzymatic activity, similar bioactivity was achieved as the wild type protein on pulmonary epithelial cells[24]. In the present study, we showed that NAMPT H247A mutant and wild type proteins afflicted the same damage to neurons both *in vivo* and *in vitro*, meaning that eNAMPT exacerbated ischemia-induced neuronal injury non-enzymatically. 

In conclusion, eNAMPT level was elevated in ischemic brain region after cerebral ischemia, which can partly be traced to peripheral blood circulation. Importantly, eNAMPT exacerbated ischemic neuronal injury non-enzymatically by triggering the release of TNF-α from glia cells. Our finding established the functional role of extracellular NAMPT protein in cerebral ischemia. Thus, neutralizing the non-enzymatic activity of NAMPT can be a novel therapeutic strategy for the treatment of cerebral ischemia, especially for patients with high eNAMPT level in serum.

## Supporting Information

Table S1 **Summary of physiological parameters before operation and after MCAO.**
(DOC)

Table S2 **Summary of physiological parameters before operation and after MCAO.**
(DOC)

Figure S1 **The effects of purified EI protein on MCAO-induced brain injury.** The rats were i.c.v. injected with 4 μl saline (NS), 4 μg EI in 4 μl saline at 12 h interval for three times with the first injection 12 h before MCAO. (**A**) Representative brain sections stained with TTC. (**B**) Statistical analysis of the infarct volume. (**C**) Neurological deficit score evaluated before sacrifice. N=6 (NS) and N=8 (EI), *P<0.05, **P<0.01, compared with NS, One-way ANOVA.
(TIF)

Figure S2 **The effect of recombinant EI protein on OGD-induced cell necrosis in neuron-glial mixed culture.** EI protein was applied 30 min before OGD. (**A**) Representative images of triple immunofluorescence staining of NeuN (green, neuron specific marker), PI (red, for necrotic cells) and DAPI (blue, for all cells) under normal condition or after OGD and recovery. (**B**) Percentage of necrotic cells in total cells (PI-positive cell number over DAPI-positive number). (**C**) Percentage of necrotic neurons in total neuron cells (PI/NeuN double positive cell number over NeuN-positive cell number). N=6. ***^##^*** *P*<0.01, ^###^ *P*<0.001, compared with the control of control, One-way ANOVA.
(TIF)
