# Supplementary material for: Cerebral Ischemia Is Exacerbated by Extracellular Nicotinamide Phosphoribosyltransferase via a Non-Enzymatic Mechanism
Source: PLoS One. 2013 Dec 31;8(12):e85403. doi: 10.1371/journal.pone.0085403 (PMC3877362; doi:10.1371/journal.pone.0085403)
Supplement: Table S1 — Summary of physiological parameters before operation and after MCAO. (DOC) [file pone.0085403.s001.doc]

Table S1. Summary of physiological parameters before operation and after MCAO

| Valuables | | Sham | MCAO  (NS *i.c.v.*) | WT  (*i.c.v.*) | H247A  (*i.c.v.*) |
| --- | --- | --- | --- | --- | --- |
| N |  | 6 | 6 | 6 | 6 |
| Body weight (g) |  | 264.8 ± 18.4 | 275.0 ± 19.0 | 273.33 ± 13.0 | 261.3± 17.26 |
| MABP (mmHg) | Before | 113.4 ± 13.1 | 108.5 ± 22.1 | 119.50 ± 16.6 | 106.6 ± 18.3 |
|  | After | 110.7 ± 18.5 | 107.1 ± 16.2 | 104.46 ± 19.9 | 102. 7 ± 17.5 |
| pO_2_ (mmHg) | Before | 102.4 ± 14.3 | 98.5 ± 12.2 | 99.15 ± 20.2 | 95.6 ± 19.8 |
|  | After | 94.3 ± 19.3 | 91.3 ± 21.1 | 92.45 ± 18.0 | 91.2 ± 16.5 |
| pCO_2_ (mmHg) | Before | 39.8 ± 13.8 | 42.1 ± 10.2 | 44.72 ± 10.6 | 40.6 ± 13.7 |
|  | After | 41.4 ± 8.3 | 38.0 ± 10.7 | 42.20 ± 17.3 | 41.4 ± 14.4 |
| pH | Before | 7.38 ± 0.22 | 7.3 ± 0.23 | 7.35 ± 0.29 | 7.35 ± 0.51 |
|  | After | 7.34 ± 0.06 | 7.4 ± 0.12 | 7.38 ± 0.17 | 7.37 ± 0.54 |
| Glucose (g/L) | Before | 6.25 ± 0.75 | 6.2 ± 1.02 | 6.18 ± 0.95 | 6.23 ± 0. 95 |
|  | After | 6.28 ± 0.69 | 6.2 ± 0.63 | 6.25 ± 0.85 | 6.27 ± 0.89 |
| rCBF (%) | Before | 100 | 100 | 100 | 100 |
|  | 10 min after ischemia | 97.8 ± 10.6 | 21.5 ± 14.1*** | 25.17 ± 17.5*** | 20.5 ± 16.4*** |
|  | 30 min after ischemia | 95.6 ± 9.3 | 20.6 ± 12.5*** | 24.82 ± 10.1*** | 21.9 ± 8.9*** |
|  | 30 min after reperfusion | 96.6 ± 3.2 | 93.5 ± 14.5 | 95.89 ± 20.6 | 92.8 ± 18.7 |

The data was presented as mean ± SD; ****P*<0.001, vs sham, analyzed by one-way ANOVA. MABP indicates mean arterial blood pressure. rCBF indicates regional cerebral blood flow.
