# Supplementary material for: Cerebral Ischemia Is Exacerbated by Extracellular Nicotinamide Phosphoribosyltransferase via a Non-Enzymatic Mechanism
Source: PLoS One. 2013 Dec 31;8(12):e85403. doi: 10.1371/journal.pone.0085403 (PMC3877362; doi:10.1371/journal.pone.0085403)
Supplement: Table S2 — Summary of physiological parameters before operation and after MCAO. (DOC) [file pone.0085403.s002.doc]

Table S2. Summary of physiological parameters before operation and after MCAO

| Valuables | | MCAO  (NS *i.c.v.*) | EI  (*i.c.v.*) |
| --- | --- | --- | --- |
| N |  | 6 | 8 |
| Body weight (g) |  | 256.2 ± 18.1 | 248.3 ± 15.3 |
| MABP (mmHg) | Before | 107. 4 ± 14.8 | 109.0 ± 16.8 |
|  | After | 107. 6 ± 16.6 | 107.6 ± 10.7 |
| pO_2_ (mmHg) | Before | 98. 0 ± 14.98 | 99.2 ±12.2 |
|  | After | 91. 4 ± 12.82 | 92.4 ± 11.8 |
| pCO_2_ (mmHg) | Before | 42. 4 ± 13.17 | 43.2 ± 10.4 |
|  | After | 37. 6 ± 12.35 | 42.1 ± 11.3 |
| pH | Before | 7.35 ± 0.21 | 7.34 ± 0.13 |
|  | After | 7.37 ± 0.14 | 7.31 ± 0.27 |
| Glucose (g/L) | Before | 6.30 ± 0.76 | 6.28 ± 0.49 |
|  | After | 6.23 ± 0.56 | 6.29 ± 0.33 |
| rCBF (%) | Before | 100 | 100 |
|  | 10 min after ischemia | 21.9 ± 16.8 | 23.3 ± 11.3 |
|  | 30 min after ischemia | 24.6 ± 15.2 | 24.2 ± 14.1 |
|  | 30 min after reperfusion | 91.7 ± 16.6 | 93.5 ± 13.0 |

The data was presented as mean ± SD; MABP indicates mean arterial blood pressure. rCBF indicates regional cerebral blood flow.
