# Supplementary figures and images for: Cerebral Ischemia Is Exacerbated by Extracellular Nicotinamide Phosphoribosyltransferase via a Non-Enzymatic Mechanism
Source: PLoS One. 2013 Dec 31;8(12):e85403. doi: 10.1371/journal.pone.0085403 (PMC3877362; doi:10.1371/journal.pone.0085403)

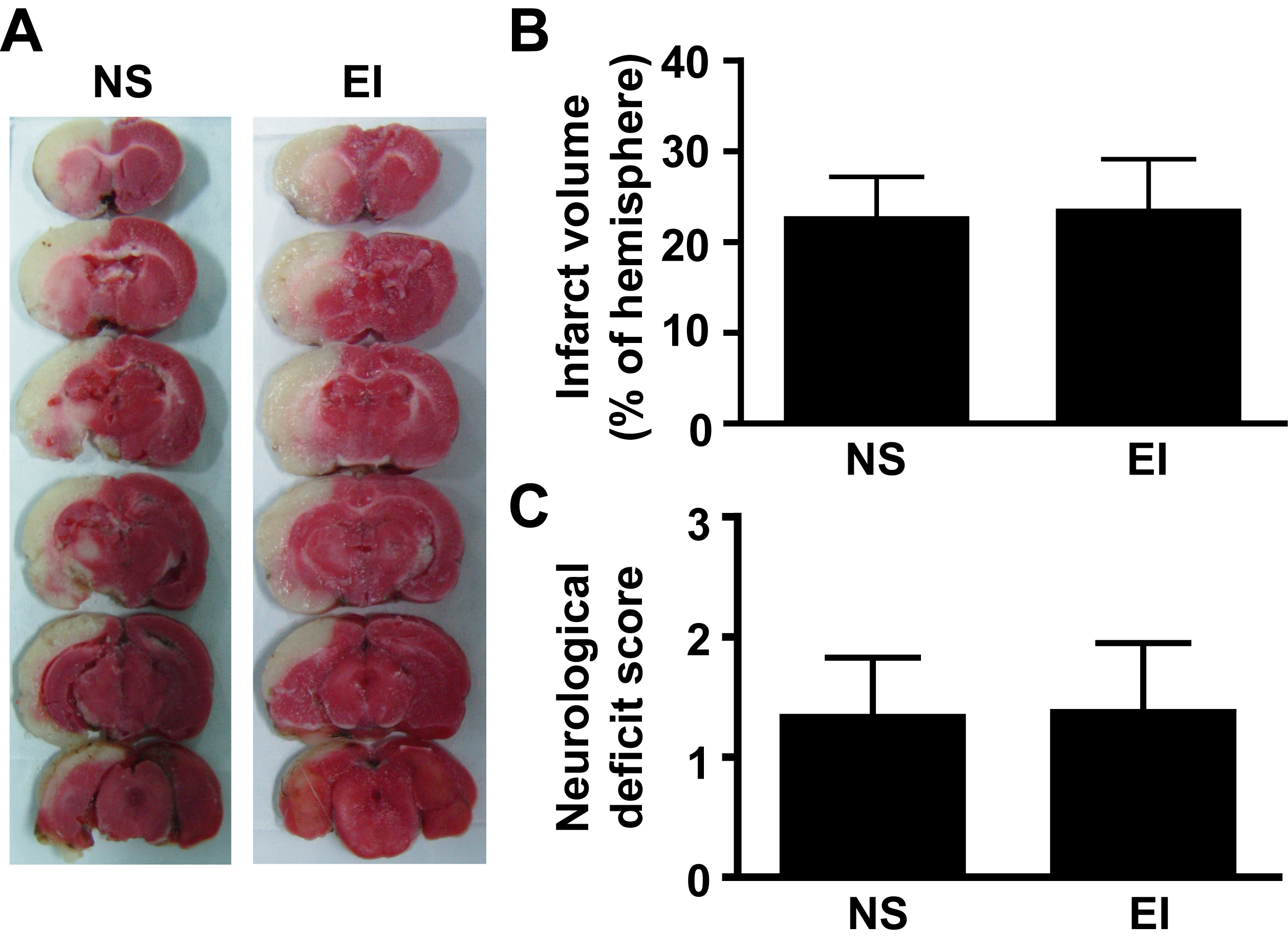

Supplement: Figure S1 — The effects of purified EI protein on MCAO-induced brain injury. The rats were i.c.v. injected with 4 μl saline (NS), 4 μg EI in 4 μl saline at 12 h interval for three times with the first injection 12 h before MCAO. (A) Representative brain sections stained with TTC. (B) Statistical analysis of the infarct volume. (C) Neurological deficit score evaluated before sacrifice. N=6 (NS) and N=8 (EI), *P<0.05, **P<0.01, compared with NS, One-way ANOVA. (TIF) [file pone.0085403.s003.tif]

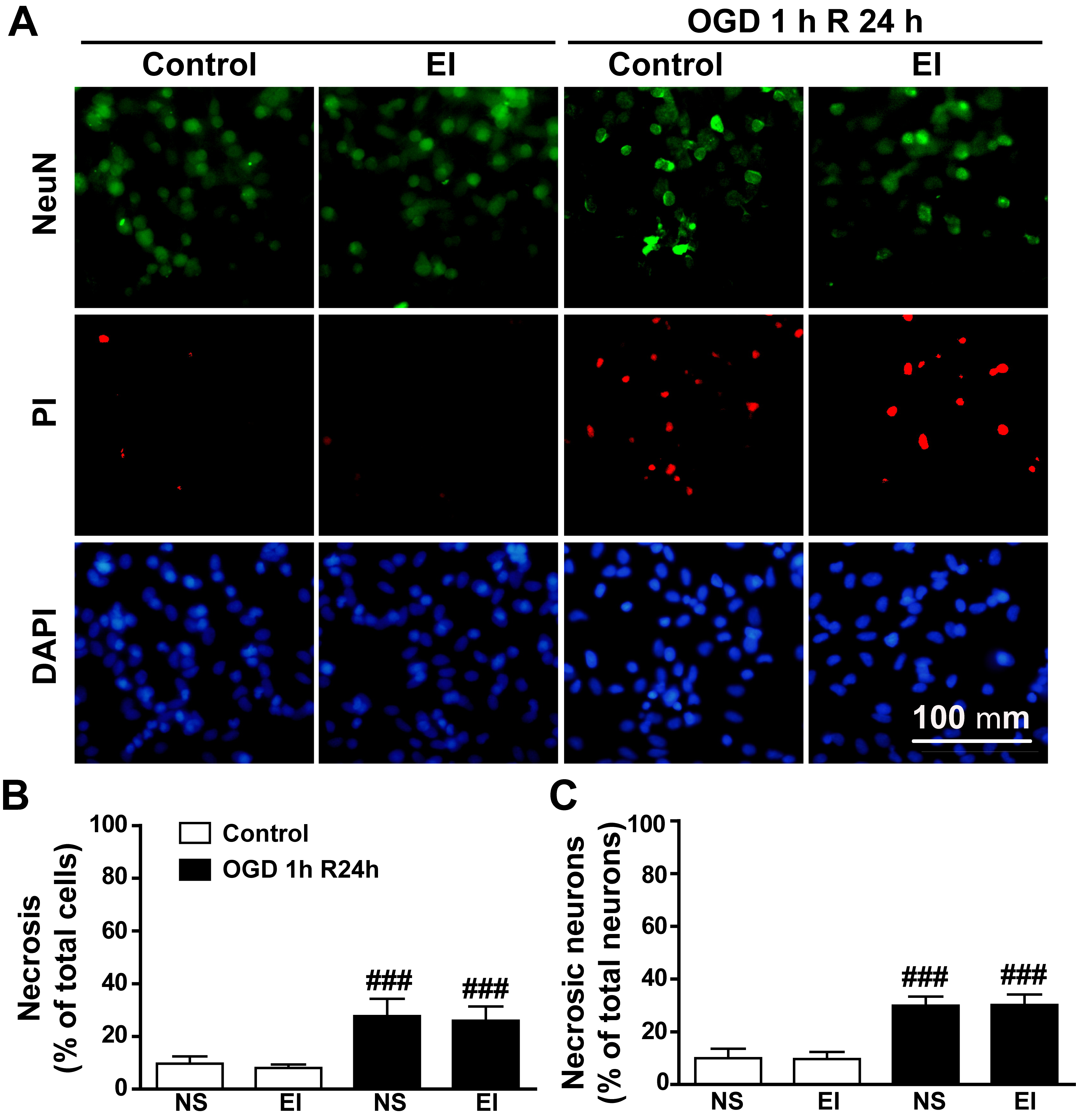

Supplement: Figure S2 — The effect of recombinant EI protein on OGD-induced cell necrosis in neuron-glial mixed culture. EI protein was applied 30 min before OGD. (A) Representative images of triple immunofluorescence staining of NeuN (green, neuron specific marker), PI (red, for necrotic cells) and DAPI (blue, for all cells) under normal condition or after OGD and recovery. (B) Percentage of necrotic cells in total cells (PI-positive cell number over DAPI-positive number). (C) Percentage of necrotic neurons in total neuron cells (PI/NeuN double positive cell number over NeuN-positive cell number). N=6. ## P<0.01, ### P<0.001, compared with the control of control, One-way ANOVA. (TIF) [file pone.0085403.s004.tif]
